# Synthesis and Anti-hypertensive Effects of the Twin Drug of Nicotinic Acid and Quercetin Tetramethyl Ether

**DOI:** 10.3390/molecules19044791

**Published:** 2014-04-16

**Authors:** Zhonglei Wang, Liyan Yang, Shuai Cui, Yingxi Liang, Xiaohua Zhang

**Affiliations:** 1 School of Chinese Materia Medica, Beijing University of Chinese Medicine, Beijing 100102, China; E-Mails: wzlei2011bzy@sina.com (Z.W.); cuishuaistrive@163.com (S.C.); yxx.liang@163.com (X.L.); 2 College of Science, China University of Petroleum, Beijing 102249, China; E-Mail: ylyan2011cup@163.com (L.Y.)

**Keywords:** rutin, nicotinic acid, synthesis, twin drug, VB3-QTME, SHR rats, anti-hypertensive effects

## Abstract

A novel twin drug consisting of nicotinic acid (VB3) and quercetin tetramethyl ether (QTME) has been synthesized as an antihypertensive in a total yield of 79.2% through methylation, hydrolysis, acylation and esterification starting from rutin. The structures of synthesized compounds were elucidated by ^1^H-NMR, ^13^C-NMR and elemental analysis. The anti-hypertensive effects of an oral daily dose (15 mg/kg) of the synthesized compounds in spontaneously hypertensive (SHR) rats and normotensive Wistar Kyoto (WKY) rats were analysed. The data demonstrate that the twin drug VB3-QTME both reduces the elevated blood pressure and prolongs the action time in SHR rats without effect on WKY rats. However, definitive evidence of a precise mechanism of action by which VB3-QTME might decrease blood pressure remains elusive. Based on the results, the therapeutic potential of this twin drug is discussed.

## 1. Introduction

Hypertension is a chronic medical condition that can cause diverse complications which may not only markedly impair the quality of life but also cause a heavy financial burden on families and society. Currently, the incidence of hypertension is the highest in the world among all diseases, affecting one-third of the global adult population [1]. As an important public-health challenge worldwide, the prevention, detection, treatment, and control of hypertension should receive high priority [2]. A majority of hypertensive patients receive two or more antihypertensive drugs, and it shows that although many patients reach blood pressure goal, combination antihypertensive therapy is often needed [3,4,5,6]. A drug with a single target cannot meet the demands of complex diseases and thus lack efficacy, although considerable progress has been made in the field of antihypertensive drugs. In fact, adverse events with monotherapy and combination therapy were as anticipated for the specific classes of antihypertensive therapy [7]. Thus, the enthusiasm still remains about the development and development of safer antihypertensive.

Western medicines show rapid and remarkable effects against hypertension, but these drugs show toxic effects clinically. Although Traditional Chinese medicine (TCM) has gained much attention clinically because of its advantages of lower toxicity and fewer side effects, protection of target organs, multiple targets, and multiple pathways, its disadvantage is that these drugs require a long time to exert their effects [8,9,10]. There are potential advantages in giving such agents with complementary pharmacological activities in the form of a single chemical entity [11,12].

Epidemiological studies have shown an inverse association of flavonoid-rich diet consumption with the risk of hypertension and cardiovascular disease [13]. Quercetin is the most common ﬂavone in the human diet, it has a wide range of reported biological effects, including antioxidant, antihypertensive, antimicrobial, and antiprotozoal activities [14,15,16,17,18]. Nicotinic acid (niacin, VB3), one of the older drugs used to treat hyperlipidemia, was shown to reduce low-density lipoprotein-cholesterol (LDL-C) and triglycerides and to markedly increase high-density lipoprotein-cholesterol (HDL-C) levels [19,20,21,22]. However, because of nicotinic acid’s acute vasodilatory effects, it also reduces blood pressure (BP), which is an cardiovascular disease risk factor [23,24,25,26,27].

In the present study, quercetin was selected for conjugation with nicotinic acid to obtain a quercetin-antihypertensive double prodrug. Although, linking of quercetin with VB3 in 1:1 ratio is difficult due to the presence of a number of hydroxyl groups, we have been able to conjugate this agent in the form of its derivative, quercetin tetramethyl ether (QTME) with VB3. In this paper, synthesis and anti-hypertensive activity of the twin drug of nicotinic acid and quercetin tetramethyl ether (VB3-QTME) are reported.

## 2. Results and Discussion

**Scheme 1:**
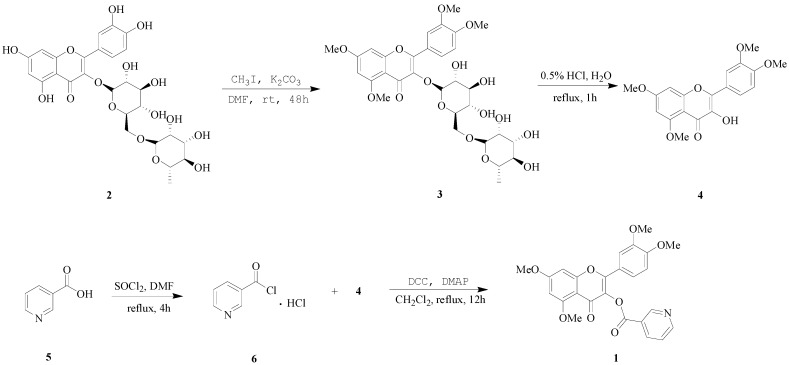
Synthetic route to VB3-QTME.

To conjugate the polyphenolic flavonoid quercetin with carboxyl group containing anti-antihypertensive drugs in 1:1 ratio, an alternative strategy was developed. For this purpose, rutin (**2**), the glycoside of quercetin was treated with methyl iodide (CH_3_I) in dry dimethyl formamide (DMF) in the presence of potassium carbonate (K_2_CO_3_). The methylated glycoside **3** was obtained as semisolid, which was subjected to hydrolysis by refluxing in acidic aqueous solution to obtain the quercetin derivative, quercetin tetramethyl ether (QTME, **4**). The free hydroxyl group generated at 3-position in this derivative was used as a synthetic handle for conjugation with the VB3 (**5**). Compound **5** was treated with thionyl chloride (SOCl_2_) by stirring at reflux and after removing excess of SOCl_2_, nicotinoyl chloride hydrochloride **6** was obtained as a white solid. QTME was treated with compound **6**, dicyclohexylcarbodiimide (DCC) and 4-dimethylaminopyridine (DMAP) using dichloromethane as solvent and the desired derivative, VB3-QTME (**1**) was obtained as light yellow solid, with a total yield of 79.2%. The sequence of various steps involved in the reactions is shown in Scheme 1.

We tested the antihypertensive effects of this VB3-QTME twin drug in sixteen-week-old SHR where the hypertension is well established. Our results showed that, while having no effect on blood pressure in control WKY rats, the VB3-QTME caused significantly more pronounced reduction in systolic blood pressure in SHR compared to combined use of VB3 and QTME. The antihypertensive effects of VB3-QTME appeared to last slightly longer than the combined use of two agents. And it is not surprising that a number of laboratories have reported that quercetin lowers blood pressure in spontaneously hypertensive [28] and Dahl salt-sensitive rats [29] as well as rats that consume a high-sucrose diet [30], are deficient in NO [31], are infused with angiotensin [32], or have experimentally induced pressure overload using aortic constriction [33]. The aforementioned animal studies have provided important proof-of-principle that quercetin may be efficacious in decreasing blood pressure. What is more, mechanism of hypotensive action of nicotinic acid remains elusive. As to VB3-QTME, potential mechanisms maybe more complex, as it could include both mechanisms of VB3 and QTME/quercetin, definitive evidence of a precise mechanism remains elusive.

## 3. Experimental

### 3.1. General Information

All commercial reagents and solvents were used as received without further purification unless specified and reactions were monitored by thin-layer chromatography (TLC) on normal-phase silica gel GF254 plates. Spots on the TLC plates were visualized using ultraviolet light (254 nm or 365 nm). Silica gel (200–300 mesh) was used as stationary phase to isolate the compounds. ^1^H-NMR and ^13^C-NMR spectra were run on Bruker Advance DPX 500- and 125-MHz spectrometers in CDCl_3_-*d* or DMSO-*d_6_*, and tetramethylsilane (TMS) was used as the internal standard. High resolution (HR)-electrospray ionization (ESI)-mass spectroscopy (MS) was measured on a Bruker APEX IV FT-MS (7.0 T) mass spectrometer in positive-ion mode. ESI-MS was obtained on an Agilent XCT 6320 IT mass spectrometer.

### 3.2. Chemistry Synthesis

#### 3.2.1. Preparation of 5,7,3',4'-*O*-tetramethylrutin (**3**)

To a fine suspension of rutin (**2**, 1.8 g, 3.0 mmol) in dry N,N-dimethylformamide (30 mL), anhydrous potassium carbonate (4.2 g, 30.0 mmol) and methyl iodide (1.9 mL, 30.0 mmol) were added and the reaction mixture was refluxed for 48 h. The solution was filtered and insoluble potassium salts were washed with acetone. The washings were combined with the filtrate and solvent was removed under reduced pressure to obtain methylated glycoside **3** as a semisolid residue.

#### 3.2.2. Preparation of Quercetin 5,7,3',4'-tetramethyl Ether (**QTME, 4**)

The above product was refluxed with aqueous hydrochloric acid (0.5%, 300 mL) for 1 h. The solvent was removed after cooling and the residue obtained was washed with distilled water to neutral to give QTME (**4**, 0.99 g, 92.2%), yellow solid, mp 195–197 °C (lit [34]: mp 194 °C). ^1^H-NMR (500 MHz, CDCl_3_) δ: 6.33 (1H, d, *J* = 2.01 Hz, C-6-H), 6.53 (1H, d, *J* = 2.01 Hz, C-8-H), 7.53 (1H, d, *J* = 8.50 Hz, C-5'-H), 7.72 (1H, dd, *J* = 8.50 Hz, *J* = 2.50 Hz, C-6'-H), 7.79 (1H, d, *J* = 2.50 Hz, C-2'-H), 9.47 (s, 1H, 3-OH), 3.87–3.97 (s, 12H, 4 × -OCH_3_). ^13^C-NMR (DMSO-*d_6_*) δ: 172.658 (C-4), 164.147 (C-9), 160.528 (C-7), 156.572 (C-2), 150.338 (C-5), 146.608 (C-4'), 142.148 (C-3'), 138.030 (C-3), 123.705 (C-1'), 120.666 (C-6'), 110.995 (C-2'), 110.341 (C-5'), 106.302 (C-1'), 96.198 (C-6), 95.555 (C-8), 56.581, 56.086, 55.831, 55.627 (4-OCH_3_). Anal. calcd. for C_19_H_18_O_7_: C, 63.68; H, 5.06; O, 31.25. Found: C, 63.81; H, 5.11. HRMS (ESI) *m/z*: 359.11804 [M+H]^+^, calcd. for C_19_H_19_O_7_ 359.11253. Spectroscopic data of the compound was consistent with those in the literatures [35,36,37].

#### 3.2.3. Preparation of Nicotinoyl Chloride Hydrochloride (**6**)

Under the anhydrous condition, nicotinic acid (8.12 mmol) and 2 drops of dry DMF were added into the vigorously stirred SOCl_2_ (10 mL) at 0 °C. The reaction mixture was heated to 78 °C to reflux for 3 h to yield the yellow solid after the excess SOCl_2_ was evaporated under reduced pressure. Then 10 mL diethyl ether was added to reflux for 1 h, and then filtered to obtain the white solid nicotinoyl chloride hydrochloride 1.388 g (yield: 96.0%).

#### 3.2.4. Synthesis of VB3-QTME (**1**)

QTME (0.050 g, 0.14 mmol) and nicotinoyl chloride hydrochloride (0.027 g, 0.15 mmol) were dissolved in dichloromethane (30 mL) containing DCC (0.173 g, 0.84 mmol) and DMAP (0.149 g, 0.84 mmol). The reaction mixture was stirred at reflux for 12 h. After reaction, the solvent was removed under reduced pressure. After washing with saturated Na_2_CO_3_ and NH_4_Cl, successively, the solid product obtained was chromatographed on silica gel column using petroleum ether/ethyl acetate/acetone/methyl alcohol (200:100:30:40) as eluent and the solvent was removed under reduced pressure to obtain light yellow needle-like crystals of VB3-QTME (**1**); yield (0.058 g, 89.5%), mp 168–170 °C. ^1^H-NMR (500 MHz, CDCl_3_) δ: 6.40 (1H, d, *J* = 2.02 Hz, C-6-H), 6.59 (1H, d, *J* = 2.02 Hz, C-8-H), 6.94 (1H, d, *J* = 2.50 Hz, C-2'-H), 7.53 (2H, ABq, *J* = 8.50 Hz, C-5'-H, C-6'-H), 9.47 (1H, s, PyC-2-H), 8.90 (1H, d, PyC-6-H), 8.47 (1H, d, PyC-4-H), 7.41 (1H, t, PyC-5-H), 3.84–3.94 (s, 12H, 4 × -OCH_3_). ^13^C-NMR (DMSO-*d_6_*) δ: 172.519 (C-4), 164.505 (C-7), 163.295 (ester C=O), 160.528 (C-5), 159.294 (C-9), 155.294 (C-2), 152.526 (PyC-3'), 151.293 ((PyC-2'), 142.148 (C-3'), 141.171 (C-4'), 138.005 (C-3), 135.028 (PyC-5'), 125.521 (PyC-1'),124.677 (PyC-4'), 121.511 (C-1'), 120.666 (C-6'), 110.995 (C-2'), 110.341 (C-5'), 108.087 (C-1'), 96.198 (C-6), 93.908 (C-8), 56.577, 56.093, 55.826, 55.622 (4 -OCH_3_). Anal. calcd. for C_25_H_21_NO_8_: C, 64.79; H, 4.57; N, 3.02; O, 27.62. Found: C, 64.83; H, 4.59; N, 2.97. HRMS (ESI) *m/z*: 464.13925 [M+H]^+^, calcd. for C_25_H_22_NO_8_ 464.13400.

### 3.3. Animal Experiments

Sixteen-week-old, male SHR and WKY rats were obtained from Weitong-Lihua Experimental Animal Technical Co., LTD (Beijing, China). All the experiments were performed in accordance with Institutional Guidelines for the ethical care of animals. All rats were maintained three per cage at a constant temperature (24 + 1 °C), with a 12 h dark/light cycle and on standard rat chow. An adaptation period of 2 weeks for vehicle administration and blood pressure measurements was allowed before the initiation of the experimental protocols.

Twenty-four SHR (six rats/group) were randomly assigned to a VB3-QTME group (15.0 mg/kg/day, 32.4 mmol/kg/day, mixed in 1 mL of 0.5% carboxymethylcellulose sodium), a quercetin group (9.8 mg/kg/day, 32.4 mmol/kg/day, mixed in 1 mL of 0.5% carboxymethylcellulose sodium), a nicotinic acid+QTME group (15.6 mg/kg/day, nicotinic acid and QTME each 32.4 mmol/kg/day, mixed in 1 mL of 0.5% carboxymethylcellulose sodium), a SHR vehicle group(1 mL of 0.5% carboxymethylcellulose sodium). Six WKY were assigned to a vehicle group (1 mL of 0.5% carboxymethylcellulose sodium), another six WKY were assigned to a VB3-QTME group (15.0 mg/kg/day, 32.4 mmol/kg/day, mixed in 1 mL of 0.5% carboxymethylcellulose sodium). During the experimental periods all the rats had free access to tap water and chow.

Before treatment, rats were trained to the procedure and measurements were recorded. Then rats were dosed once daily by oral gavage with 32.4 mmol/kg of test compounds on each of the 7 days. Systolic blood pressures were measured 4 hours after the oral gavage on each of the 7 days in awake rats by the tail-cuff method (Softron BP-98A, Softron, Beijing, China). At least six determinations were made in every session and the mean of the middle four values was taken as the systolic blood pressure level. At the end of the last day’s treatment, tail systolic blood pressures were then continuously measured in conscious rats in the following 4 h, 8 h, 12 h, 24 h, 48 h, 72 h.

**Figure 1:**
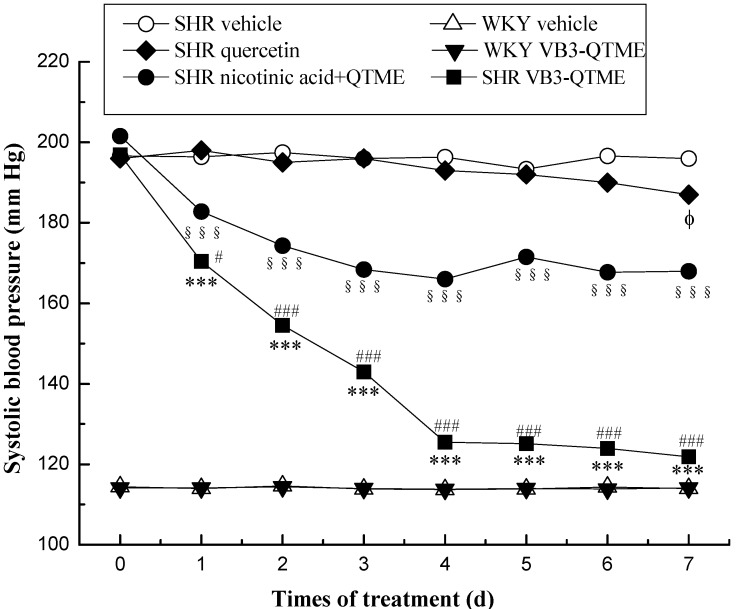
SBP in SHR and WKY rats before and after treatment. Antihypertensive effect of test compounds (SHR VB3-QTME, 15.0 mg/kg/day; SHR nicotinic acid+QTME, 15.6 mg/kg/day; SHR vehicle; WKY VB3-QTME, 15.0 mg/kg/day; WKY vehicle) during continuous feeding in rats. Each point represents the mean SBP in six rats. The data were given as means ± SEM. Comparison between experimental groups were made by using one-way ANOVA, followed by student Newman Keuls test. *p* values < 0.05 were considered significant (SPSS (PASW) 17.0). *** *p* < 0.001, between before and after VB3-QTME treatment; ^§§§^ *p* < 0.001, between before and after nicotinic acid+QTME treatment; ^ϕ^ *p* < 0.05, between before and after quercetin treatment; ^#^ *p* < 0.05, ^###^ *p* < 0.001, SHR VB3-QTME compared to SHR nicotinic acid+QTME.

**Figure 2:**
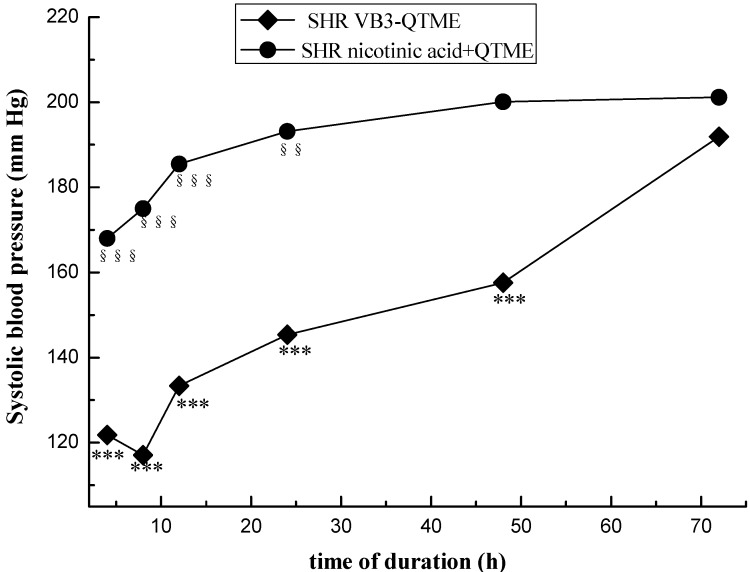
Duration time after the 7 days’ VB3-QTME and nicotinic acid+QTME treatment in SHR. Each point represents the mean SBP in six rats. The data were given as means ± SEM. *** *p* < 0.001, between before treatment and after the 7 days’ treatment VB3-QTME; ^§§^ *p* < 0.01, ^§§§^ *p* < 0.001, between before treatment and after the 7 days’ treatment nicotinic acid+QTME.

The data were given as means ± SEM. Comparison between experimental groups were made by using one-way ANOVA, followed by student Newman Keuls test. *p* values < 0.05 were considered significant (SPSS (PASW)17.0). Systolic blood pressures of SHR and Wistar rats before and after treatment are presented on Figure 1. Systolic blood pressures of SHR after the 7 days’ treatment VB3-QTME or nicotinic acid+QTME are presented on Figure 2.

Figure 1, ● represents the changes in tail systolic blood pressures of SHR orally administered nicotinic acid+QTME (each 32.4mmol/kg), and it was observed that the blood pressure remarkably declined after administration (^§§§^ *p* < 0.001). ◆ represents the changes in tail systolic blood pressures of SHR with quercetin(32.4mmol/kg), and it was observed that the blood pressure declined after 7 days’ administration (^ϕ^ *p* < 0.05). ■ represents the changes in tail systolic blood pressures of SHR orally administered VB3-QTME (32.4mmol/kg), and it was observed that the blood pressure remarkably declined after administration (*** *p* < 0.001). However, the VB3-QTME group exhibited more significant (^#^ *p* < 0.05 on the first day, ^###^ *p* < 0.001 on the next other days) reduction effects on blood pressure.

Figure 2, ◆ represents the changes in tail systolic blood pressures of SHR with VB3-QTME, it was observed that the maximal reduction of blood pressure at the eighth hour after the 7 days’ administration, and the anti-hypertensive activity of VB3-QTME can last 48 h (*** *p* < 0.001). ● represents the changes in tail systolic blood pressures of SHR with nicotinic acid+QTME, it was observed that the maximal reduction of blood pressure at the fourh hour after the 7 days’ administration, and the anti-hypertensive activity of VB3-QTME can last 24 h (*** *p* < 0.001). However, the VB3-QTME group exhibited longer duration effects on blood pressure.

Herein we demonstrated that treatment with nicotinic acid+QTME for 7 days (15.6 mg/kg/day, each 32.4 mmol/kg/day) produced an around 15%-reduction in systolic blood pressure, and the treatment with VB3-QTME for 7 days (15.0 mg/kg/day) almost normalized systolic blood pressure. Similar to our data, other study in SHR documented that treatment with quercetin for 7 days (10.0 mg/kg/day) produced a 10%-reduction in mean blood pressure [38]. Another study in SHR documented that treatment with quercetin for five weeks (10 mg/kg/day) reduced blood pressure around 21%, but had no effect in normotensive animals [39]. In addition, data from the literature indicates that there is no difference between the oral administration of quercetin as a single or divided into two daily doses [40].

Evidence exists to support several potential mechanisms whereby quercetin might decrease blood pressure and decrease the severity of hypertension in animals and humans. These mechanisms are a decrease in oxidative stress, interference with the renin-angiotensin-aldosterone system (RAAS), and/or improving vascular function in an endothelium-dependent or -independent manner [41,42,43,44,45]. As to VB3-QTME, potential mechanisms maybe more complex, as it could include both mechanisms of VB3 and QTME/quercetin, definitive evidence of a precise mechanism remains elusive. Furthermore, it needs to be determined whether VB3-QTME is an effective treatment for all forms of hypertension regardless of pathological origin. However, despite the uncertainty of the mechanism of action of VB3-QTME, it has promise for the treatment of hypertension.

## 4. Conclusions

Herein, an efficient and convenient synthesis of VB3-QTME consisting of VB3 and QTME has been achieved by a four-step reaction starting from the readily available nicotinic acid and rutin through methylation, hydrolysis, acylation and esterification in 79.2% yields is presented. The anti-hypertensive activity of VB3-QTME was recorded, and the data demonstrate that this twin drug both reduces the elevated blood pressure and prolongs the action time in SHR rats without effect on WKY rats. Based on the results, the exact mechanism of VB3-QTME and its applications are yet to be explored further.
